# Gaussian entanglement generation from coherence using beam-splitters

**DOI:** 10.1038/srep38002

**Published:** 2016-11-28

**Authors:** Zhong-Xiao Wang, Shuhao Wang, Teng Ma, Tie-Jun Wang, Chuan Wang

**Affiliations:** 1 State Key Laboratory of Information Photonics and Optical Communications, School of Science, Beijing University of Posts and Telecommunications, Beijing 100876, P. R. China; 2 State Key Laboratory of Low-Dimensional Quantum Physics and Department of Physics, Tsinghua University, Beijing 100084, P. R. China

## Abstract

The generation and quantification of quantum entanglement is crucial for quantum information processing. Here we study the transition of Gaussian correlation under the effect of linear optical beam-splitters. We find the single-mode Gaussian coherence acts as the resource in generating Gaussian entanglement for two squeezed states as the input states. With the help of consecutive beam-splitters, single-mode coherence and quantum entanglement can be converted to each other. Our results reveal that by using finite number of beam-splitters, it is possible to extract all the entanglement from the single-mode coherence even if the entanglement is wiped out before each beam-splitter.

Quantum correlation, especially quantum entanglement, is a key ingredient in quantum information science. Since the Einstein-Podolsky-Rosen (EPR) paradox has been put forward^1^, quantum correlation has been investigated both in theory and in experiments^2^,^3^,^4^,^5^,^6^,^7^,^8^. During the past decades, the characterization and definition of quantum correlation have attracted much research interest, such as Bell non-locality, quantum steering, quantum entanglement, quantum discord, and quantum coherence^9^,^10^,^11^,^12^,^13^,^14^,^15^,^16^,^17^. It has been pointed out that these definitions satisfy hierarchy relations, i.e., quantum coherence > quantum discord > quantum entanglement > quantum steering > Bell non-locality^18^,^19^,^20^,^21^, where *A* > *B* represents *B* is a subset of *A*, meaning all steerable states are entangled, but not all entangled states have steering, etc. On the other hand, quantum entanglement characterizes the nonclassical property of multipartite quantum systems. An entangled state can not be decomposed as a convex sum of product states^14^, if two subsystems are entangled, the quantum state of each subsystem can not be described independently. Quantum entanglement only represents the nonclassical property between the subsystems which can not characterize the nonclassical property of a single-party system. Non-classicality can be used to characterize its nonclassical property of the quantum states, such as squeezed states, anti-bunched states, and sub-Poissonian states^22^. The non-classicality characterizes the quantumness of a single-party quantum system. The concept of quantum steering comes from the EPR paradox. For a quantum system consisting of subsystems marked with *A* and *B, A* can steer *B* if *A* can affect *B*’s subsystem by using a local operation on *A*’s subsystem. Quantum steering has many useful applications, such as sub-channel discrimination, one-side device-independent quantum key distribution, etc^23^,^24^,^25^,^26^.

In addition to quantum correlation, quantum coherence can also be used to characterize the nonclassical property of both multipartite and single-party quantum systems. Quantum coherence is one of the key features of quantum mechanics, several experiments have revealed that quantum coherence exists in photosynthetic complexes and indicating that it plays a key role in high efficiency achievements in photosynthesis^27^,^28^. Quantum coherence can be quantified by the off-diagonal elements in the density matrix of the system under the reference basis, which is called the *l*_1_ norm coherence. Another way to measure coherence is the relative entropy coherence defined as the entropy difference between the density matrix of the quantum state and the diagonal density matrix that eliminating all the off-diagonal elements of the original density matrix.

Quantum systems can be roughly partitioned into discrete and continuous variable ones. As the most important family of continuous quantum states, Gaussian states are widely used because of its availability and controllability in experiments^29^. Recently, some representative applications have been investigated, such as quantum communication, ultrasensitive sensing, detection and imaging, and so on ref. 30. Different from discrete variable quantum states^13^,^14^,^15^, the approaches of quantifying the quantum correlation of continuous variable quantum states are more complicated^12^,^31^,^32^. Here in this study, we will give a detailed mathematical expression of Gaussian correlation and investigate the Gaussian correlation generated by beam-splitters in experiments. Beam-splitters are key components in optics, and have been used in many experiments including Bell test experiment, Wheeler’s delayed choice experiment, etc^33^,^34^. Especially in quantum information processing, Knill *et al*. pointed that efficient quantum computation is possible to be realized by using only beam-splitters, phase shifters, single photon sources and photo-detectors^35^. Although beam-splitters are linear optical devices, they can generate quantum entanglement^36^,^37^,^38^,^39^.

In this work, we study the behavior of the quantum correlation of Gaussian states under the effect of beam-splitters. We find that the non-classicality of single-mode Gaussian state generates entanglement by beam-splitters^40^ is not valid for all single-mode Gaussian states. Instead, single-mode coherence plays a vital role in such process. By using consecutive beam-splitters, we find the single-mode coherence and quantum entanglement could be converted to each other for two squeezed states as the input states. Moreover, if we further wipe out the entanglement before each beam-splitter, all the quantum entanglement can be extracted from the single-mode coherence by a finite number of beam-splitters.

## Results

### Entanglement and steering generation using single beam-splitter

Here we consider two single-mode Gaussian optical beams send into the two input ports of one beam-splitter. The covariance matrix of the output Gaussian optical beam can be characterized by


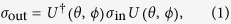


where *σ*_out(in)_ is the covariance matrix of the output (input) optical beam, *U(θ, ϕ*) could be described by^40^


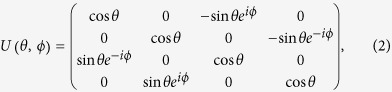


where 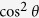 denotes the transmittance and *ϕ* represents the phase difference between the reflected and transmitted fields. Here we consider the case where two single-mode input Gaussian optical beams are squeezed lights, one (say mode *A*) is squeezed in position quadrature and the other one (say mode *B*) is squeezed in momentum quadrature. The covariance matrices of modes *A* and *B* are given by


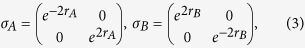


where *r*_*A(B*)_ is the squeezing parameter for mode *A* (*B*). The covariance matrix of the output optical beam becomes *σ*_out_ = *U*^†^(*θ, ϕ*) (*σ*_*A*_⊕*σ*_*B*_)*U* (*θ, ϕ*).

The quantum steering of the output optical beams could be calculated by


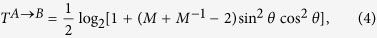


where 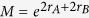. It is obvious that quantum steering can be created by the beam-splitter except the case where *r*_*A*_ = −*r*_*B*_ (meaning two optical beams are identical) or *θ* = *nπ*/2 (*n* is an integer). By considering the case 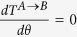, the maximal value of quantum steering is found at the point *θ* = (2*n* + 1)*π*/4. And the quantum entanglement of the output state could be obtained by


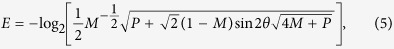


where 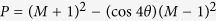.

**Figure 1:**
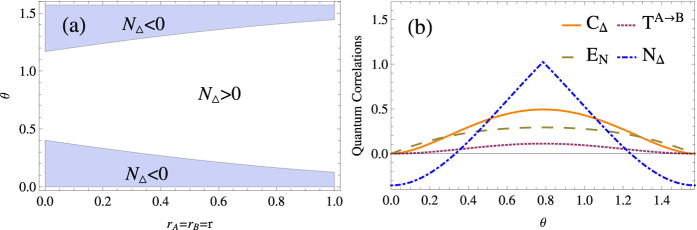
The quantum correlation of the Gaussian optical beams undergoing a beam-splitter, where the input states are two squeezed lights. (**a**) Represents the difference of the single-mode non-classicality between input *N*_in_ and output *N*_out_ optical beams where *r*_*A*_ = *r*_*B*_ = *r*. (**b**) Represents the change of the quantum correlation when *r*_*A*_ = *r*_*B*_ = 0.2, where *C*_Δ_ is the difference of the single-mode coherence between input and output optical beams, *E*_*N*_ is the quantum entanglement of the output beam, *T*^*A*→*B*^ is the quantum steering of the output beam.

In the following, we will study the change of the non-classicality of the two single-mode Gaussian states after the action of a beam-splitter. By using


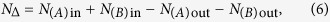


one can find *N*_Δ_ exhibits the similar behavior as quantum entanglement. In a previous work^40^, the authors have found that the non-classicality creates entanglement in the case where the input states are nonclassical Gaussian states and vacuum states. Here we do not use these states as the input states because mixing these two states by a beams splitter can not generate quantum steering. However, when the two input single-mode Gaussian states are two squeezed lights, their finding is not valid since *N*_Δ_ ≥ 0 does not always satisfied. Here Fig. 1(a) describes the region where *N*_Δ_ < 0 (*r*_*A*_ = *r*_*B*_ = *r*), and Fig. 1(b) displays the transition of quantum correlation when we regard *θ* as an independent variable and assume that *r*_*A*_ = *r*_*B*_ = 0.2. One may find that when 0 < *θ* < 0.33 and 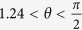, both quantum steering and quantum entanglement increase and the non-classicality also increase. Therefore, quantum entanglement and quantum steering are not generated by single-mode non-classicality.

Then we consider single-mode coherence as


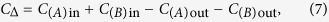


we find the relation *C*_Δ_ ≥ 0, and *C*_Δ_ equals to zero only when *r*_*A*_ = −*r*_*B*_ or *θ* = *nπ*/2. Meanwhile, *C*_Δ_ shows similar behavior as quantum entanglement and quantum steering. Here we numerically study the change of single-mode coherence in Fig. 1(b), where we choose *θ* as an independent variable and *r*_*A*_ = *r*_*B*_ = 0.2. Since *C*_in_ keeps as a constant with a fixed *θ*, when *C*_out_ = *C*_(*A*)out_ + *C*_(*B*)out_ decreases, both quantum entanglement and quantum steering increase, meaning that they can be generated by single-mode coherence. Here we can conclude that quantum entanglement between two-mode Gaussian states can be regarded as an intrinsic coherence, i.e., coherence between the two modes. The intrinsic coherence and single-mode coherence are complementary to each other. In other words, intrinsic coherence increases along with the decrement of single-mode coherence^41^. According to the definition^42^, quantum correlations originates from the superposition of quantum states. The single-mode coherence can characterize the superposition of the subsystem (the local superposition), the intrinsic coherence (or quantum entanglement) represents the superposition between the subsystems (the unlocal superposition). Our observation indicates that the beam-splitters can be used to convert local superposition to nonlocal superposition, and vice versa.

### Conversion between entanglement and quantum steering using Gaussian optical beams

**Figure 2:**
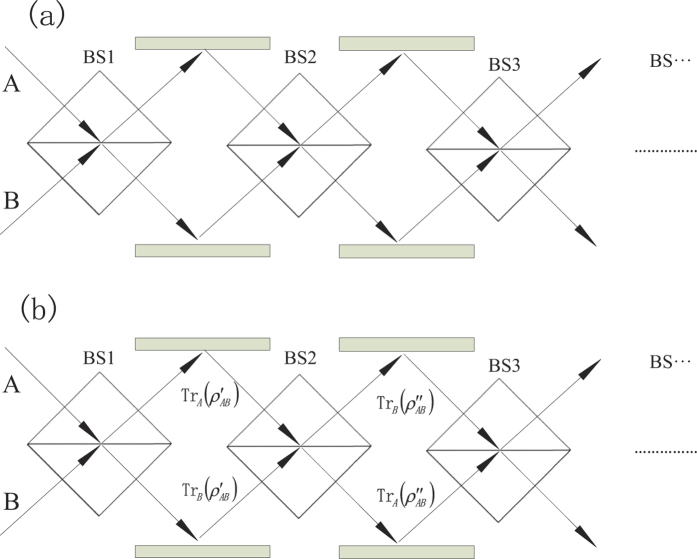
Schematic picture of two optical beams going through consecutive beam-splitters (BS), where (**a**) represents entanglement is not wiped out, (**b**) represents entanglement is wiped out.

**Figure 3:**
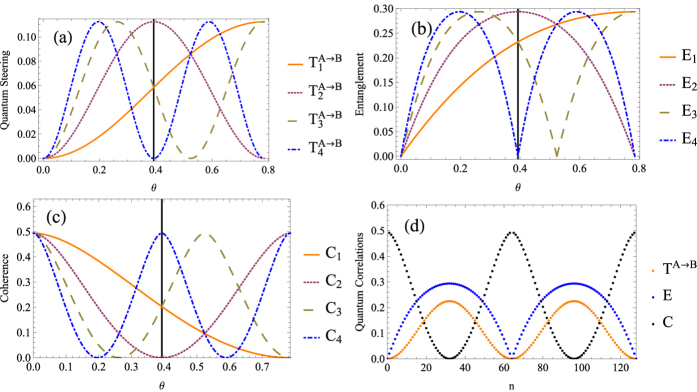
The quantum correlation of the output beams when *r*_*A*_ = *r*_*B*_ = 0.2, where *C* = *C*_*A*_ + *C*_*B*_. (**a**) The quantum steering, (**b**) the quantum entanglement, and (**c**) the quantum coherence represent the case where 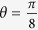. (**d**) Represents the case where 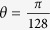. The subscripts represent the number of beam-splitters.

We study in the following the case when more than one beam-splitters are used to mix the squeezed lights. From Eq. (2), we find that *U(θ, ϕ*) is a periodic function by considering *θ* as an independent variable. By choosing the input optical beams at the beam-splitters with 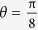 (see Fig. 2(a)), we find both quantum entanglement and quantum steering increase after the first and second beam-splitters, and decrease when undergoing the third and fourth ones while quantum coherence shows the opposite behavior. Here in Fig. 3(a,b,c), we plot the evolution of quantum steering, quantum entanglement and quantum coherence for different number of beam-splitters as *n* = 1, 2, 3, 4, respectively. It is obvious that under the effect of consecutive beam-splitters, quantum entanglement (quantum steering) and quantum coherence convert to each other. Figure 3(d) illustrates the evolution of quantum correlation when *θ* = *π*/128 and *r*_*A*_ = *r*_*B*_ = 0.2. In this case, the evolution of the quantum correlation behaves like a non-Markov process without loss. Therefore, with the memory effect, consecutive beam-splitters can be used to simulate the non-Markovian environment.

**Figure 4:**
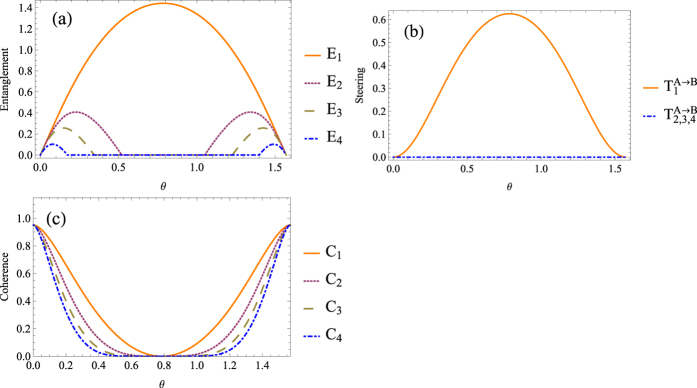
The (**a**) quantum entanglement, (**b**) quantum steering, and (**c**) single-mode coherence of the output beam when quantum entanglement being wiped out before consecutive beam-splitters, where *r*_*A*_ = *r*_*B*_ = 0.5 and *C* = *C*_*A*_ + *C*_*B*_, the subscripts represent the number of beam-splitters.

To obtain the condition under which quantum entanglement is wiped out before the input optical beams and further mixed by the next beam-splitter, as illustrated by Fig. 2(b), we find that the quantum entanglement of the input beams after the last beam-splitter will decrease with the increment of the number of the beam-splitters. And the number of beam-splitters depends on *θ*, for instance, when 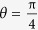, quantum entanglement can only be generated by the first beam-splitter. When *θ* → 0, quantum entanglement can always be generated. Figure 4 shows the quantum entanglement, quantum steering and single-mode coherence when considering *n* = 1, 2, 3, 4. The quantity of the generated entanglement depends on single-mode coherence of the input states. Once quantum entanglement has been erased, it can not convert back into single-mode coherence, and the observed entanglement will decrease with respect to *n*. On the other hand, quantum steering can only be generated by the first beam-splitter regardless of *θ* (see Fig. 4(b)). Since quantum entanglement has been wiped out before the consecutive beam-splitters, we can conclude that the process could be observed during which quantum entanglement is converted to quantum steering.

## Summary

We have studied the progress of quantum entanglement and quantum steering generation using beam-splitters. We have found that instead of the single-mode Gaussian non-classicality, single-mode Gaussian coherence acts as the fundamental resource for generating two-mode Gaussian entanglement and steering for two squeezed states as the input states. The covariance matrix of general one-mode Gaussian states can be written as 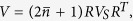 where *V*_*S*_ is the covariance matrix of squeezed states, 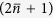 is a constant, *R* represents the phase rotation operator. By further calculations, we find when the two input squeezed states have the same phase rotation, the conclusion is also correct. Therefore, we suppose the conclusion is valid for general one-mode Gaussian states. Based on the former results, it has been confirmed by the observation that single-mode coherence and the entanglement are complementary to each other, namely, quantum entanglement increases while single-mode coherence decreases, and vice versa.

Meanwhile, we have discussed the evolution of quantum entanglement under the action by several beam-splitters on two single squeezed states, and discovered that quantum entanglement and single-mode coherence transfers to each other periodically. If we wipe out the entanglement in the consecutive beam-splitters, only the first beam-splitter generates quantum steering. We hope these results on quantum correlation could further develop the applications in quantum information processing.

## Method

### Definition of Gaussian states

The continuous variable quantum system (Gaussian state) is always difficult to be characterized by using the density matrix since the dimensionality of the density matrix is infinite. Here we consider the canonical operators in the vector by 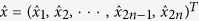, where 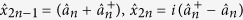, and 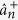 (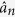) are the creation (annihilation) operators for the *n*-bosonic mode system. The elements in 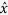 satisfy the commutation relation as 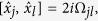 where 
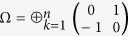
 is denoted the symplectic form.

The covariance matrix *σ* contains the elements of which is defined by^29^


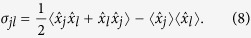


Here the diagonal elements of *σ* are the variance of 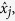 and the off-diagonal elements encode the inter-modal correlations among subsystems. And the quantity 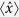 is defined as the mean value of 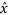. The physical meanings of 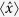 characterizes the center of the probability distribution in phase space and *σ* describes its shape.

The covariance matrix of a two-mode Gaussian state consisting of parties *A* and *B* could be expressed as


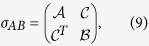


where 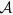 and 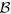 are the covariance matrices for subsystems *A* and *B*. And 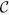 characterizes the correlation between *A* and *B*.

### Quantum correlation of Gaussian states

The definition of negativity characterizes by the entanglement of two-partite discrete variable systems^14^,^15^. Similar to discrete variable condition, we use logarithmic negativity to characterize the entanglement of Gaussian states, which can be calculated by *E* = max{0, −log_2_*v*}, where *v* is the smallest symplectic eigenvalue of the partial transpose of the covariance matrix. This definition also originates from the positive partial transpose (PPT) criteria^15^,^31^. In this work, we consider two-mode Gaussian states, the partial transpose is defined as


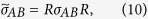


where *R* = diag{1, −1, 1, 1}, and *σ*_*AB*_ denotes the covariance matrix of the two-mode Gaussian state.

Quantum steering is also a type of quantum correlation whose definition is located between quantum entanglement and Bell non-locality^12^. It describes how local operations on one subsystem effects another. Assuming that a two-mode Gaussian state consisting of subsystems *A* and *B, A* can steer *B* by *A*’s Gaussian measurements if the condition *σ*_*AB*_ + *i*(0_*A*_ ⊕ Ω_*B*_) ≥ 0 is violated^43^, where Gaussian measurements consists a measurement set that maps Gaussian states into Gaussian ones. The quantum steering from *A* to *B* can be obtained by


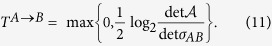


The definition above is only suitable for two-mode Gaussian states, consisting of subsystems *A* and *B*, by implementing Gaussian measurements on *A*’s subsystem. In this paper, we use Eq. (11) to calculate the quantum steering of two-mode Gaussian states.

The non-classicality represents the nonclassical property of the non-classical states, such as squeezed states, anti-bunched states, and sub-Poissonian states whose correlation function can not be reproduced by any classical field^22^. The non-classicality of the Gaussian states can be characterized by the covariance matrix of the system. For single-mode Gaussian states, some quantities (for example, the degree of squeezing) are used to characterize the non-classicality. In this paper, we use *N* = −log_2_*λ*_min_ to calculate the non-classicality^40^, where *λ*_min_ is the minimum eigenvalue of the covariance matrix (ref. 40 gives the equation *N* = −log_2_2*λ*_min_ since they defined 
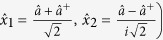
.

Quantum coherence characterizes the superposition property of quantum systems. The relative entropy coherence for discrete variable systems can be written as ref. 13


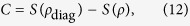


where *S(ρ*) = −Tr(*ρ*log_2_*ρ*), and *ρ*_diag_ denotes the diagonal density matrix that eliminating all the off-diagonal elements of *ρ* under the reference basis. In this paper, we use the coherence measure as 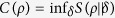, where *δ* is the nearest incoherent Gaussian state of *ρ*. The coherence of one-mode Gaussian states is ref. 32:


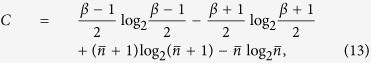


where 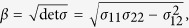 and 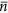 denotes the mean value of the thermal state number, which can be calculated by 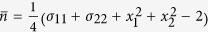, where *x*_*i*_(*i* = 1, 2) are the components of 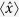. In this study, we choose *x*_*i*_ = 0 and we have 
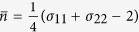
.

## Additional Information

**How to cite this article**: Wang, Z.-X. *et al*. Gaussian entanglement generation from coherence using beam-splitters. *Sci. Rep.* **6**, 38002; doi: 10.1038/srep38002 (2016).

**Publisher's note:** Springer Nature remains neutral with regard to jurisdictional claims in published maps and institutional affiliations.
